# Indium and Silver Recovery from Perovskite Thin Film Solar Cell Waste by Means of Nanofiltration

**DOI:** 10.1021/acssusresmgt.5c00109

**Published:** 2025-05-16

**Authors:** Meret Amrein, Karina Rohrer, Dirk Hengevoss, Heon Jin, Henry J. Snaith, Michael Thomann, Frank Nüesch, Markus Lenz

**Affiliations:** † Institute for Ecopreneurship, School of Life Sciences, University of Applied Sciences and Arts Northwestern Switzerland, Hofackerstrasse 30, 4132 Muttenz, Switzerland; ‡ EPFL, Institute of Materials Science and Engineering, Ecole Polytechnique Fédérale de Lausanne, Station 12, Lausanne 1015, Switzerland; § Empa, Swiss Federal Laboratories for Materials Science and Technology, Laboratory for Functional Polymers, Dübendorf, 8600, Switzerland; ∥ Department of Environmental Technology, Wageningen University, 6708 PB Wageningen, The Netherlands; ⊥ Department of Physics, 6396 University of Oxford, Clarendon Laboratory, Parks Road, Oxford OX1 3PU, United Kingdom

**Keywords:** perovskite solar cell recycling, element recovery, advanced membrane filtration, resource supply criticality

## Abstract

Due to minimal material use and low-cost processing, next-generation thin film solar cells represent a promising alternative to traditional crystalline silicon solar cells. Among these, metal-halide perovskite solar cells have seen significant improvements in power conversion efficiency and are now on the verge of market entry. However, most efficient and stable perovskite solar cells contain lead in the perovskite absorber layer, along with indium and silver in their electrodes. This study demonstrates an environmentally benign recycling process for recovering all three elements from end-of-life perovskite solar cells. In short, the process consists of mechanical dismantling (milling), aqueous extraction/purification of PbI_2_, and acid extraction and purification of indium and silver by nanofiltration. After the quantitative recovery of lead as PbI_2_ (95 ± 5%), indium and silver were dissolved using nitric acid with recovery rates of 87 ± 7% for both metals. Life cycle assessment calculations were used to determine optimal conditions in terms of minimal environmental impact per gram of extracted element. After acid extraction, nanofiltration was employed using both custom-made layer-by-layer membranes and commercially available acid-resistant flat sheet membranes to separate indium from silver. Using an optimized membrane design, indium was almost entirely retained (96.9 ± 0.4%) using a layer-by-layer membrane at 50% permeate recovery. Hence, a twofold concentration of indium was achieved over the course of the filtration. In contrast, silver was not retained (retention of −7.6 ± 6.3%), resulting in a dilute Ag permeate. Using the commercial flat sheet membrane resulted in similar retention rates, with 98.5 ± 0.4% for indium and 5.8 ± 11.6% for silver. However, this came at the expense of considerably higher operating pressure (25 bar vs 5 bar) and lower flux (6 L/m^2^h vs 30 L/m^2^h), resulting in higher energy demand (72 Wh/L vs 9 Wh/L). Therefore, layer-by-layer membrane filtration proved to be the superior method for element recovery from perovskite photovoltaic devices. This study has shown that combining hydrometallurgical processing (aqueous and acidic extraction) with layer-by-layer membrane filtration offers an efficient and environmentally benign approach for metal recovery from end-of-life solar cells. Since indium and silver are also key elements for other thin film photovoltaic applications, layer-by-layer membrane filtration may represent a platform technology for future photovoltaic panel recycling.

## Graphical abstract

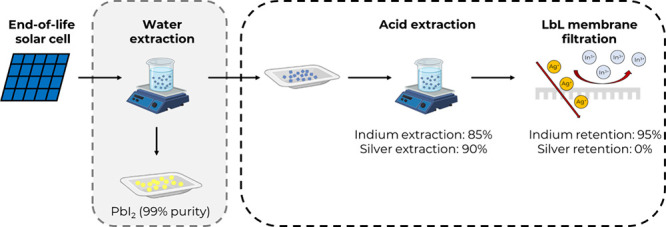

## 1 Introduction

With the declaration of the European Green Deal, which aims to achieve climate neutrality by 2050, and in the face of climate change threatening the well-being of humanity, the demand for renewable energy sources is steadily increasing.^1^ Solar energy is now amongst the cheapest renewable energies and covers a large share of the growing demand.^2–4^ Most of the photovoltaic (PV) devices currently installed are crystalline silicon (Si) solar cells.^5^ However, thin film solar cell technologies including perovskite solar cells (PSCs) have gained tremendous interest in recent years.^6^ One reason for this interest is that, within just a few years of development, PSCs have achieved power conversion efficiencies comparable to those of established Si solar cells.^7^ Due to the high absorption coefficient of perovskite semiconductors, the material use is considerably lower compared to crystalline Si, positioning PSCs as a promising low-cost alternative.^8^ Moreover, perovskite precursors can be solution processed, enabling scalable and high-throughput fabrication methods, such as roll-to-roll printing, including slot-die coating or chemical vapor deposition.^9–11^ It can be expected that Si-perovskite tandem solar cells may be the first perovskite technology to enter the market at high volumes.^12^

Most of the highly efficient and stable PSCs reported contain valuable metals such as silver (Ag) and indium (In), along with potentially toxic materials like lead (Pb).^13,14^ The supply of Ag and In is a primary concern, particularly given the expected increase in installed PV capacity in the coming years.^15^ The demand for Ag is rising, not only for PV applications but also due to the increasing complexity of electronic devices and its exceptional electrical conductivity.^16^ Similarly, In is widely used as a component of indium tin oxide (ITO) for applications that require transparent electrodes. Furthermore, In has been classified as critical raw material (CRM) in many countries, exhibiting a high supply risk, especially since future demand is expected to increase.^13,15^ Due to the limited abundance of both elements and the anticipated rise in demand for various electronic devices, In and Ag are considered limiting resources for perovskite PV technology.^14^ Therefore, recycling these elements at the end-of-life of solar cells is crucial for ensuring raw material supply and environmental protection.^17^ Solar panels also fall under the Waste Electrical and Electronic Equipment (WEEE) directive, which mandates the sustainable collection and recycling of waste electronic products in the European Union.^18–20^ Similar regulations exist in other countries, such as the Regulations for the Administration of the Recovery and Disposal of Waste Electrical and Electronic product in China or the Resource Conservation and Recovery Act (RCRA) in the United States, which regulates the disposal of hazardous waste based on leaching potential, determined via the so called Toxicity Characteristic Leaching Procedure (TCLP).^21,22^

Currently widely applied and standardized processes for PV recycling include mechanical disassembly and recycling of aluminum frames and junction boxes, solar cell delamination (i.e., removal of the polymeric encapsulation), as well as the separation and recovery of the delaminated glass.^23^ Metallurgical recycling of valuable materials such as silver or silicon is currently rarely applied on an industrial scale, but due to increasing material scarcity and PV production, new recycling technologies are emerging.^24^ Most (pilot) projects focus on high purity Ag leaching and Si wafer purification for solar cell reuse.^24^ Thin film PV technologies make up about 5% of the total PV market. A recycling process to recover 95% of the semiconductor material of CdTe solar cells has been developed.^25^ In contrast, PSCs are just entering the PV market, and large-scale PSC recycling has not been implemented so far.^24^ However, several recovery techniques for In and Ag, based on hydrometallurgical and/or pyrometallurgical methods, have been reported.^26–28^ Hydrometallurgical methods offer advantages such as flexible separation processes, higher energy efficiency, and lower associated emissions.^29,30^ As a result, most proposed processes for recycling In and Ag from PV systems rely on hydrometallurgy.^31^ Zhang et al. proposed a two-step process for recovering In and Ag from crystalline Si PV, using 10 wt % NaOH for Ag extraction, followed by In extraction using 6 M H_2_SO_4._ ^32^ Teknetzi et al. successfully extracted In and Ag from end-of-life copper-indium-gallium-selenide (CIGS) cells using 2 M HNO_3_ over 24 h.^33^ Quantitative Ag recovery from incinerated organic PV cells was achieved with 1.4 M HNO_3._ ^34^ In contrast, recycling In and Ag from PSCs has been rarely reported. A few wet chemical methods have suggested selective dissolution of the active solar cell layers.^35,36^ However, the use of high amounts of organic solvents, including problematic ones like dimethylformamide (DMF), makes these approaches difficult to implement on an industrial scale.^35,36^

Subsequent purification and recovery of metals from acidic extracts typically rely on classical hydrometallurgical methods such as solvent extraction, ion exchange, and/or precipitation.^31^ Nanofiltration (NF) is an emerging technology for element recovery that can either replace and/or complement traditional hydrometallurgical approaches,^37,38^ as it relies on a different physicochemical separation principle (i.e., size and charge of ions).^37^ In NF, a charged active layer introduces the repulsion of solutes with increasing charge, leading to the retention of multivalent ions (e.g., the metal ion of interest), while uncharged and monovalent ions (i.e., impurities and non-dissociated acid) can pass through the membrane.^39^ Acid-resistant NF has been used for both acid purification and the recovery of dissolved metals (see, for example, refs 37, 40, and references therein); however, full-scale implementation is currently limited by long term stability issues or high energy consumption due to the high operating pressures required for filtration.^39,41^

Despite rapid technological advancements, few options exist for Ag and In extraction and purification that are both technically feasible and environmentally sound. This study therefore investigated the acidic extraction of In and Ag from end-of-life PSCs, followed by the separation and concentration of the In and Ag enriched acidic stream by using layer-by-layer (LbL) membrane filtration. This work builds upon previous research on recycling PbI_2_, which successfully recovered all Pb as pure PbI_2_, leaving a solid residue not classified as hazardous according to the US Toxicity Characteristic Leaching Procedure.^42^ The optimal extraction conditions for Ag and In from the solid residue were determined based on elemental yields (measured by triple quadrupole inductively coupled plasma mass spectrometry, QqQ-ICP-MS) and associated environmental impacts (determined by life cycle assessment (LCA)). After acid extraction, the elements were separated and concentrated by using NF with commercially available flat sheet membranes and custom-made LbL NF membranes. Optimal purification conditions were determined by balancing rejection, flux, and the required power consumption for filtration.

## 2 Methods

### 2.1 Analytical Methods

All metal analyses were performed on an ICP-MS system (8800 QqQ-ICP-MS, Agilent, Basel, Switzerland) using general-purpose operational settings. Quantification was performed via external calibration from multielement standards using ^103^Rh^+^ as an internal standard to account for matrix effects. For extraction experiments, ^208^Pb^+^, ^107^Ag^+^, ^115^In^+^, and ^118^Sn^+^ were used for quantification, and the ICP-MS was operated by using helium as a collision gas (5 mL/min). HNO_3_ (67%, trace metal grade, Sigma-Aldrich, Basel, Switzerland) was used for sample acidification.

### 2.2 Extraction Experiments

A synthetic PSC mixture was prepared based on a common PSC composition^11^ (Table S1). In this process, only the electrode materials (i.e., ITO and Ag) were considered for extraction since the formamidinium lead iodide (FAPbI_3_) layer was fully removed by aqueous extraction.^42^

Synthetic PSC extraction was carried out over 24 h at room temperature, 60 °C, and 80 °C. The experiments were performed in 20 mL batches using a reaction station for simultaneous heating and stirring (Carousel 12 plus, reaction station, Radleys, UK). ITO nanopowder and Ag foil (all Sigma-Aldrich, Basel, Switzerland) were dissolved in HNO_3_ at concentrations of 1%, 3%, 5%, and 10%, as well as in deionized water. The amounts of ITO and Ag were based on typical material thicknesses in the PSC (Table S1). The volume of acid for extraction corresponded to 1 or 2 L per m^2^ of the solar cell.

Extraction of glass perovskite stacks (ITO/PEDOT:PSS/FACsPbSnI_3_/C60/BCP/Ag, 3 × 3 cm) was done without any prior size reduction. PbI_2_ recycling, based on previous work, was conducted prior to the acidic extraction experiments, which were carried out on the solid residue after PbI_2_ recycling.^42^ Based on the sample area, the extraction acid (5% HNO_3_) volume was calculated using an area-equivalent concentration of 1 or 2 L per m^2^ of the solar cell.

### 2.3 Nanofiltration Membranes

Poly­(ether sulfone) (PES)-based hollow fiber ultrafiltration (UF) membranes, provided by Pentair (Enschede, the Netherlands), were used as a support structure to prepare LbL membranes. The membranes were potted in modules containing one fiber each. Each hollow fiber had an inner diameter of 0.8 mm and a length of 300 mm, resulting in a total membrane surface area of 7.5 cm^2^ per module. After potting, the bare membranes were immersed in deionized water overnight. The application of polyelectrolytes on the membranes was carried out using a custom-made setup that enabled dynamic coating, wherein dead-end filtration was used to concentrate the polyelectrolytes inside the lumen of the membrane.^40^ Positively charged polyallylamine (PAH; MW = 65 kDa, 10 wt % in water) and negatively charged poly­(sodium styrenesulfonate) (PSS; MW = 1000 kDa, 25 wt % in water) were purchased from Sigma-Aldrich (Basel, Switzerland). The oppositely charged polyelectrolytes were diluted in a NaCl solution (0.5 M) and were alternately coated onto the membrane, starting with the positively charged PAH and always terminated by PSS. The pH of the coating solution was neutral. After each coating cycle, the membrane was flushed with deionized water until the conductivity fell below 7 μS/cm. The conductivity was measured using a GMH 3451 conductivity meter from Greisinger (Regenstauf, Germany).

An acid-resistant flat sheet membrane (AMS NanoPro A-3012) with a membrane area of 200 cm^2^ was purchased from Unisol Membrane Technology (Gotha, Germany). The membrane was immersed in deionized water overnight before experiments.

### 2.4 Membrane Filtration

Model solutions for filtration experiments were based on the In and Ag concentrations found in the synthetic PSC extraction experiments using optimized conditions (In = 1000 mg/L, Ag = 1000 mg/L). For this, In­(NO_3_)_3_ (99.99% trace metal basis, Sigma-Aldrich, Basel, Switzerland) and AgNO_3_ (99.99% trace metal basis, Sigma-Aldrich, Basel, Switzerland) were dissolved in concentrated HNO_3_ and diluted to a final concentration of 5 wt % acid. When necessary, the pH of the feed solutions was adjusted using NaOH (40, reinst, Carl Roth, Karlsruhe, Germany). The pH was measured using a WTW inoLab Multi 9620 IDC instrument (Huberlab, Aesch, Switzerland).

LbL membranes were tested in a custom-made filtration device (Figure S4).^40^ At a transmembrane pressure (TMP) of 5 bar, a BVP-Z gear pump (Ismatec, Switzerland) was used to establish the desired cross-flow velocity. At a TMP of 5 bar, the flow was 160 mL/min, resulting in a cross-flow velocity of 2.65 m/s and a Reynolds number of >2300, indicating turbulent flow. Each LbL membrane filtration experiment was performed in triplicate.

The commercial flat sheet nanofiltration membranes were tested in a modular filtration unit (MaxiMem, PS Prozesstechnik, Switzerland). Experiments were conducted at a cross-flow flux of 5 L/min and a pressure of 25 bar. All filtration experiments were performed at room temperature.

### 2.5 Life Cycle Assessment

The global warming potential (GWP 100) per gram of extracted In and Ag was calculated by an LCA conducted with the SimaPro software and the Ecoinvent v3 database. The LCA in this study was performed following the ISO 14040:2006 Standard (ISO, 2006), which includes the following steps: (1) definition of goal and scope, (2) life cycle inventory (LCI), (3) life cycle impact assessment (LCIA), and (4) interpretation (details in Section S2).

## 3 Results

### 3.1 Acidic Extraction of Indium and Silver

Quantitative extraction (87 ± 7%) of In and Ag was achieved after 24 h at 80 °C for all tested acid concentrations (Figure 1). A high (>85 ± 10%) Ag extraction was already observed after 1 h of extraction, whereas the dissolution of In showed concentration dependent extraction kinetics: when using 10% HNO_3_, 80% In dissolution was observed after 7 h of extraction, (Figure 1A), while only around 50% dissolution was observed when using 5% HNO_3_ at the same time (Figure 1B). The extraction kinetics did not increase when the acid volume was doubled to 2 L/m^2^ solar cell (Figure 1C). At room temperature and 60 °C, the extraction kinetics were slow, and quantitative dissolution of In and Ag was only possible using 10% HNO_3_ and >72 h for extraction (Figure S1). Extraction of the minute amounts of Sn (from ITO, see SI) was low, and the concentrations in extracts were under the limit of quantification. Thus, Sn was not considered further in the current study.

**Figure 1:**
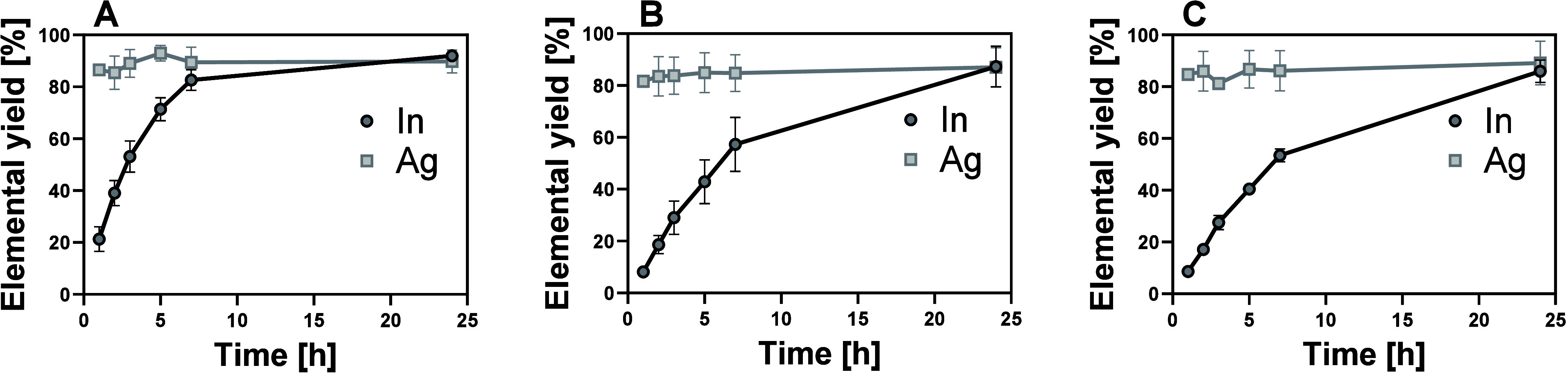
Acid extraction of a synthetic PSC mixture using 10% HNO_3_ and an area equivalent of 1 L/m^2^ (A), 5% HNO_3_ and an area equivalent of 1 L/m^2^ (B), and 5% HNO_3_ with an area equivalent of 2 L/m^2^ (C). Experiments were conducted in triplicate at 80 °C.

### 3.2 Separation and Concentration of Indium and Silver Using Nanofiltration

The highest In retention (96.3 ± 0.02%) was observed using a PES­(PAH/PSS)_4_ membrane at pH 1 (Figure 2). A lower retention (89.6 ± 0.01%) was observed at pH 0, which corresponded to the pH of the unchanged feed solution after PSC extraction. Further increasing the pH to pH 2 did not lead to an additional increase in In retention. Likewise, In retention was low when using a PES­(PAH/PSS)_3_ membrane but significantly increased to 95–99% when an additional polyelectrolyte bilayer was added. The coating of more than four bilayers did not result in any further improvement in filtration performance (Figure 2C). Ag retention was not significantly influenced by the number of bilayers or by changes in pH. Thus, PES­(PAH/PSS)_4_ operated with a feed at pH 1 was determined to be optimal for the current application.

**Figure 2:**
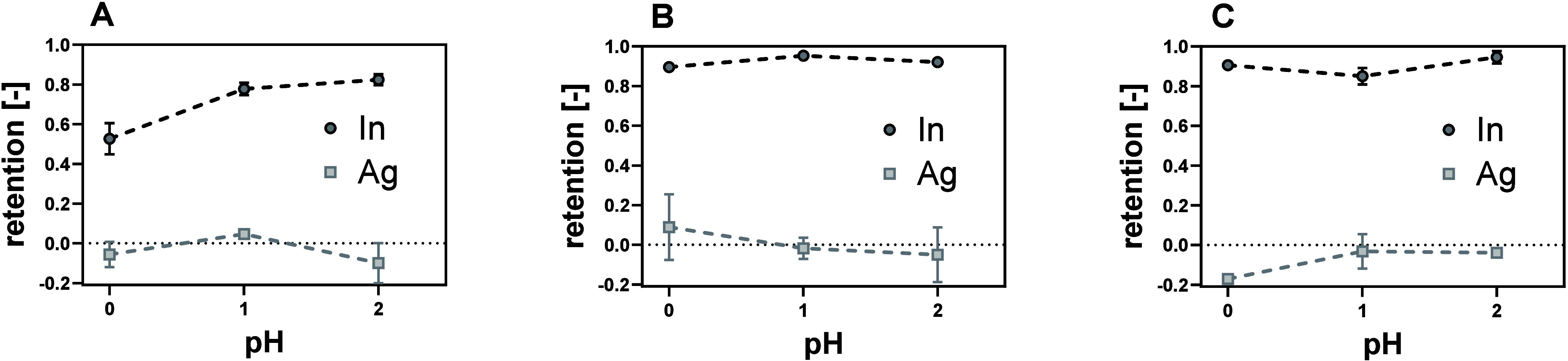
Retention of In and Ag from a synthetic PSC acid extract using 3 (A), 4 (B), and 6 (C) bilayers of PES­(PAH/PSS)_*x*_ membranes. Experiments were conducted in triplicate at 80 °C.

Further experiments showed stable In and Ag retentions at 50% permeate recovery. In retention remained high (96.9 ± 0.4%), resulting in a nearly twofold increase in In concentration (1355 ± 155 to 2089 ± 135 mg) over the course of the filtration (Figure 3A,B). In contrast, the Ag retention was slightly negative (−7.6 ± 6.3%), leading to a slight decrease in Ag concentration in the feed (714 ± 72 to 634 ± 39 mg). Comparable results have also been achieved using the commercial flat sheet NF membrane, where In retention remained high at 98.5 ± 0.4% and Ag retention was low at 5.8 ± 11.6% (Figure 3A). However, the pressure required for filtration was 25 bar, which is five times higher than that used for LbL filtration, while the flux was approximately three times lower (Figure 3C).

**Figure 3:**
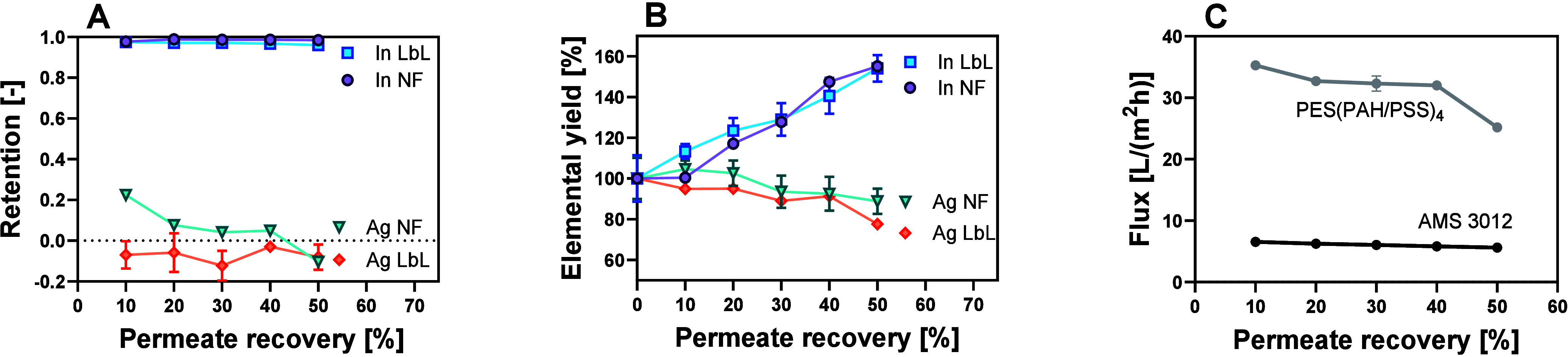
Comparison of the retention (A), metal concentration (B), and flux (C) of a PES­(PAH/PSS)_4_ LbL membrane and an AMS 3012 NF membrane over the course of filtration.

## 4 Discussion

### 4.1 Acidic Extraction of Indium and Silver

Acidic extraction of In and Ag from end-of-life PSC was evaluated by LCA, with the goal of maximizing metal extraction while minimizing environmental impact (kg_CO2‑eq_/kg_Ag or In_, using GWP 100) (Section S2). Preliminary extraction experiments at room temperature and 60 °C showed lower extraction kinetics and yields for both Ag and In (Figure S1), wherefore only extraction at 80 °C was considered for LCA calculations (Figure 1). In practice, industrial waste heat could be used for extraction, as it is often available in high amounts (heat potential in EU ∼ 300 TWh/year), which could further reduce the impacts assumed here.^43^ Comparing extraction with 5% and 10% HNO_3_, LCA calculations have shown that there is a considerable difference in GWP between Ag and In extraction. Ag was nearly quantitatively extracted after just 5 h of extraction for both acid concentrations (Figure 1A,B). Consequently, the GWP was nearly doubled for Ag extraction with 10% HNO_3_ (Figure 4A). In contrast, the GWP for In extraction was very similar for both acid concentrations since In extraction kinetics were considerably higher when using 10% HNO_3_ (Figure 4B).

**Figure 4:**
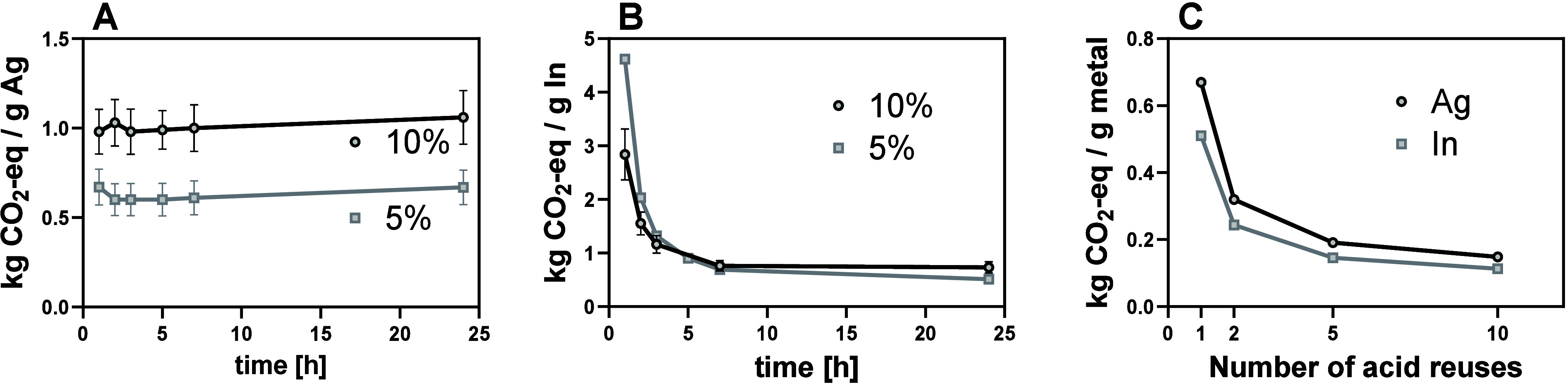
Global warming potential (GWP 100) in kg of CO_2_-eq of acidic extraction. Extraction of Ag (A), extraction of In (B), and development of GWP 100 for extraction with 5% HNO_3_ for several extraction cycles (C).

Overall, extraction using 1 L/m^2^ 5% HNO_3_ resulted in the lowest GWP. However, preliminary extraction experiments with glass-based solar cells have resulted in considerably lower extraction compared to the extraction of a synthetic PSC (Figure S2). Due to the high mass and volume of the glass substrate, extraction occurred at very high pulp densities (1300–1500 g/L), and complete wettability of the cell was not possible. Therefore, an increased acid volume may be necessary for efficient In and Ag extraction. However, it was observed that the GWP associated with the HNO_3_ used was high. As such, it will be crucial to explore possibilities for reduction of the acid’s environmental impact. Most importantly, acid reuse should be considered, especially when extraction is conducted with higher acid volumes. It was observed that the GWP could be considerably reduced when extracting Ag and In from more than 1 m^2^ of solar cell with the same amount of acid volume, where the GWP could be up to 5× lower when 1 L of acid was used for the extraction of 10 m^2^ of solar cell (Figure 4C). Thereby, the use of a higher acid volume (e.g., 2 L/m^2^ PSC) would also be possible, ensuring good wettability of the cell. However, it should be noted that for a later NF step, the membrane performance (i.e., in retention and flux) might decrease when the ionic strength in the feed increases.^44^ Optimization of the process to achieve maximal In and Ag extraction while maintaining good filtration performance will hence be crucial for scaling up the process. Furthermore, it was observed that the relative impact of heating the extraction acid increased with the number of extraction cycles. Hence, extraction at lower temperature but higher acid concentration may be more effective and should be considered for process optimization during potential scenarios.

When using plastic-based PSCs, a considerable volume reduction upon incineration of the end-of-life cells prior to acidic extraction could be an option. Søndergaard et al. demonstrated that acid consumption for extraction of Ag from end-of-life OPVs could be reduced by a factor of 100 when the cells were incinerated prior to extraction.^34^ However, possible volatilization of elements (e.g., residual iodine) and the formation of solid Ag/In metal phases that are more resistant to acid extraction from ashes need to be considered.^34^ Ultimately, it remains to be proven if an overall abatement of emissions can be achieved through prior methods (through cradle-to-cradle LCA).

### 4.2 (LbL) Nanofiltration for Concentration and Separation of In and Ag

Generally, NF membranes have shown to exhibit high retention for multivalent species and low retention for monovalent and neutral species.^40^ The effective separation between In and Ag observed here (Figures 2 and 3) is in accordance with thermodynamic equilibrium modeling. The model confirmed that In is predominantly present as multivalent In^3+^ and In­(NO_3_)^2+^, while Ag is expected to be present as monovalent Ag^+^ and neutral AgNO_3_(aq) in the current acidic conditions (Figure S3). No considerable change in speciation was observed with changing pH. A slight increase in In^3+^, alongside a decrease in In­(NO_3_)^2+^, was observed upon a pH increase from 0 to 1. This could partly explain the higher In retention at increased pH due to higher charge repulsion of the trivalent species.

LbL membrane filtration was optimized to achieve maximal In/Ag separation. At low pH, the LbL coating layers are expected to swell, resulting in increased permeability.^45^ It has been shown that increasing the pH leads to higher ion retention.^46^ In this study, neutralization to pH 1 was sufficient to obtain nearly quantitative In retention, with no further improvement observed at higher pH levels. In addition to the pH of the feed solution, the number of bilayers is also known to strongly influence ion retention. Increasing the number of bilayers may result in increased ion retention due to increased charge repulsion and decreased pore size.^47^ In this study, maximal In retention was achieved using a PES­(PAH/PSS)_4_ membrane, with no further increase in In retention observed when additional bilayers were applied. Therefore, the PES­(PAH/PSS)_4_ membrane was determined to be the most suitable LbL NF membrane for this application and was used in all subsequent experiments.

When compared to commercially available NF membranes, LbL NF membranes have the advantage of exhibiting high permeabilities while requiring low operating pressures.^40^ As a result, energy consumption can be significantly reduced.^38^ The power consumption of a membrane filtration pump depends linearly on the feed cross-flow and the applied pressure (eq 1, Section S3).^48^ Hence, power consumption increases with higher cross-flow and pressure. Additionally, the resulting energy consumption depends on the time required for filtration, i.e., the transmembrane flux (eq 2, Section S3).^38,49^ Thus, a high flux results in a lower energy consumption for filtration. In this study, we compared the energy consumption of the used LbL membrane [PES­(PAH/PSS)_4_] to that of a commercial flat sheet NF membrane (AMS 3012). Since a meaningful comparison was only possible with equal membrane areas, assumptions for potential scale-up scenarios were made. The energy consumption needed for the filtration of 1 L of feed was calculated for an estimated membrane area of 200 cm^2^ (Figure 5A) and 1 m^2^ (Figure 5B). To account for uncertainties in scale-up, a confidence range for the feed cross-flow as well as the transmembrane pressure and flux was included. However, pilot-scale experiments would be needed for a more accurate determination of the power consumption.^50^ Still, in both scenarios, the energy consumption of the LbL membrane filtration was considerably lower, mainly due to a lower operating pressure and higher flux, pointing out the potential of the LbL technology (Figure 5).

**Figure 5:**
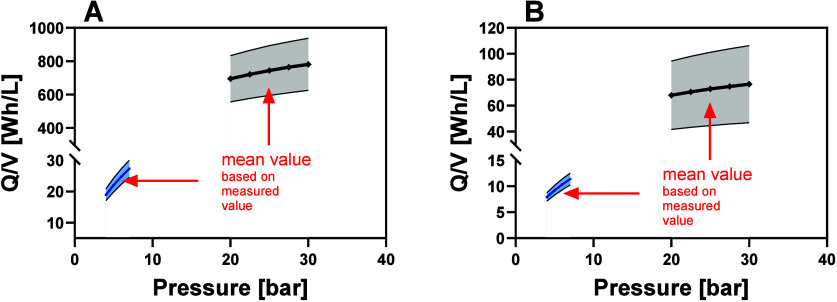
Comparison of the energy consumption for the filtration of 1 L of feed with an LbL membrane (PES­(PAH/PSS)_4_ (depicted in blue) and a commercial NF membrane (AMS 3012) (depicted in gray). The calculation was based on an estimated membrane area of 200 cm^2^ (A) and 1 m^2^ (B).

LbL NF membranes are known to be unstable when operated in highly saline streams.^44^ Chen et al. described swelling of the LbL multilayer in saline solution, which is mainly a result of dissociation due to competition between salt ions and paired polyelectrolytes.^44^ Previous studies have shown that dilution of highly saline feed solutions was necessary to ensure stable LbL filtration.^46^ Furthermore, LbL NF membranes are also expected to suffer from instability when used in highly acidic solutions.^45^ Therefore, the use of conventional NF membranes may be more advantageous than that of LbL NF membranes when operating in highly acidic solutions with high ionic strength. However, the current waste stream exhibited a relatively low ionic strength, making it suitable for LbL NF membrane applications. Additionally, the use of mild acidic conditions (5% HNO_3_, pH 1) did not result in any decline in membrane performance during the LbL NF experiments, indicating good membrane stability over 50% permeate recovery. Nevertheless, the investigation of LbL membrane stability during long-term use for the current application will be considered in future research.

Classical hydrometallurgical routes use solvent extraction, ion exchange, and/or precipitation to concentrate and purify elements of interest.^51^ Importantly, NF is based on a fundamentally different separation principle (charge and size selectivity) and can hence be applied for the purification and concentration of mixtures that are otherwise difficult to separate.^52^ Removal of impurities by NF was shown to increase the selectivity of solvent extraction (SX) as well as improve phase separation, along with reduced chemical consumption for SX.^37,53^ Additionally, the purity of the recovered metal could be higher due to the removal of impurities by NF.^38^

In and Ag recovery after filtration can be achieved by precipitation, either as chloride or through electroprecipitation.^28,54^ In precipitation can be achieved using NH_4_OH.^29^ In this context, NF is expected to positively influence the process in terms of chemical consumption and GWP. Due to the high volume reduction of the retentate stream, the NH_4_OH consumption could be considerably decreased (Section S4). Additionally, acid reuse after filtration and precipitation of In and Ag should be considered to reduce the impact of HNO_3_ on the recycling process. As nitric acid is present as monovalent (NO_3_^–^) or neutral (HNO_3_) species, it is not retained by the NF membrane and is therefore equally distributed in the permeate and retentate stream. Here, reuse of the permeate stream is expected to be more feasible than reuse of the retentate. The precipitation of Ag using HCl or electroprecipitation does not alter the pH of the solvent, making acid reuse feasible. In contrast, In precipitation using NH_4_OH results in an increase in pH, by which HNO_3_ is neutralized and rendered unsuitable for direct reuse.^29^ However, since the In-containing retentate becomes highly concentrated during filtration, the permeate accounts for >80% of the original volume fraction. Thus, concentration of the In stream enables a considerably lower acid loss of 10–20%.

### 4.3 Scarcity of Raw Materials and the Contribution of Recycling to Security of Supply

Global installed PV capacity has grown exponentially over the past 20 years and is expected to continue increasing. A global capacity of >50 TW is predicted to be installed by 2050, compared to just over 1 TW today.^14,15^ However, as the number of installed PV panels increases, the use of materials and the amount of PV panel waste will also grow.^55^ Furthermore, the demand for highly efficient PV technologies is increasing, as material use and the cost per watt of electricity produced decrease with increasing efficiency.^56^ The power conversion efficiencies of first-generation Si solar cells are set to stagnate at between 25 and 27%, driving high demand for new, highly efficient PV technologies.^57,58^ Thus, fabrication of a tandem cell using a Si bottom cell and a perovskite top cell represents one of the most promising approaches for increasing PV panel efficiency, as recent efficiency records of >34% have been reported.^59–61^ The large-scale production of Si-perovskite tandem PV is expected to grow substantially in the coming years.^14,62^ Consequently, the demand for In and Ag is also expected to increase.

The criticality of In and Ag is expected to increase for several reasons. In the case of In, its primary application is as a transparent electrode composed of ITO for flat-panel displays, and its demand increases concurrently with increasing demand for PV applications.^6,63^ Ag is used in various fields, and its high economic value increases its criticality.^17,16^ Furthermore, with the anticipated growth of PV production, the demand for In and Ag is expected to exceed current global production by 2040.^13,14^ Therefore, supply will become especially critical in the European Union since the largest market share for primary production is in China for In (80%) and South and Central America for Ag (around 50%).^64^ As the declaration of In and Ag as critical raw materials becomes increasingly likely with rising demand, the pressure to recycle end-of-life PV panels will increase, especially since the Critical Raw Materials Act mandates the recycling of at least 15% of the EU’s annual consumption by 2030.^17,65^ In the EU, the end-of-life recycling rate (EOL-RR) for In is currently 0%, highlighting the importance of developing an efficient recycling process.^66^ For Ag, the EOL-RR is considerably higher with 55%.^66^ However, the contribution of Ag recycling to the actual material need, the so called EOL recycling input rate (EOL-RIR), is only 19%, indicating a growing demand.^66^ This also demonstrates that while recycling can significantly contribute to a circular economy, reducing or replacing limiting resources like In and Ag remains crucial. Furthermore, the materials will be trapped during the lifetime of the solar cells (i.e., >25 years) and will not be available for recycling during that period.^15^ Therefore, recycling from other In- and Ag-containing secondary sources should also be prioritized to prevent a potential supply shortage. In-containing flat-panel displays, in particular, represent a promising secondary source, as In is currently not recycled on a large scale.

## 5 Conclusions

As a highly efficient and low-cost alternative to conventional silicon PV, PSCs are on the verge of entering the photovoltaic market.^67,68^ Achieving circularity through the recycling of valuable and scarce metals, such as In and Ag, is crucial to guarantee high sustainability as well as social acceptance of the technology. The recycling of highly valuable and potentially rare metals is particularly important not only for enabling circularity in next-generation PV but also for ensuring a stable resource supply in the future.

In this study, we presented a proof-of-concept process for the recovery of In and Ag from end-of-life PSCs. Acid-resistant NF using an LbL membrane has proven to be suitable for the recovery of the metals of interest from an acidic PSC extract. Our results demonstrated that LbL NF membranes exhibit good separation performance for In and Ag by quantitatively retaining In and having 0% Ag retention. Thus, prepurification and concentration of the respective waste streams were achieved, enabling a significant reduction in chemical use for the final recovery of In and Ag. LbL NF has also proven to be superior to conventional NF since comparable In/Ag separation was achieved at considerably lower energy consumption for filtration.

While the approach is promising, further evaluation of membrane acid stability and nitric acid reuse will be important for scale-up.

## Supplementary Material



## Notes

Composition of the used PSC, acidic extraction results of glass-based PSC and synthetic PSC mixtures, life cycle assessment calculations, In and Ag speciation diagrams, scheme of the used filtration units, pressure dependent energy consumption calculations, and calculations of In and Ag precipitation after NF (PDF)

## Abbreviations

PV: photovoltaic
Si: silicon
PSCs: perovskite solar cells
Ag: silver
In: indium
Pb: lead
ITO: indium tin oxide
WEEE: Waste Electrical and Electronic Equipment
RoHS: Restriction of Hazardous Substances
RCRA: Resource Conservation and Recovery Act
TCLP: Toxicity Characteristic Leaching Procedure
NF: nanofiltration
LbL: layer-by-layer
LCA: life cycle assessment
FAPbI_3_: formamidinium lead iodide
PES: poly­(ether sulfone)
UF: ultrafiltration
PAH: polyallylamine
PSS: poly­(sodium styrenesulfonate)
TMP: transmembrane pressure
GWP: global warming potential
LCIA: life cycle impact assessment
EOL-RR: end-of-life recycling rate
EOL-RIR: end-of-life recycling input rate
