# Supplementary material for: Indium and Silver Recovery from Perovskite Thin Film Solar Cell Waste by Means of Nanofiltration
Source: ACS Sustain Resour Manag. 2025 May 16;2(6):1087–95. doi: 10.1021/acssusresmgt.5c00109 (PMC12207669; doi:10.1021/acssusresmgt.5c00109)
Supplement: Supplementary file 1 [file rm5c00109_si_001.pdf]

Supporting Information for “**Indium and silver recovery from perovskite thin film solar cell waste by means of nanofiltration**”

Meret Amrein[a,b], Karina Rohrer[a], Dirk Hengevoss[a], Heon Jin[e], Henry J. Snaith[e], Michael Thomann[a], Frank Nüesch[b,c], Markus Lenz[a,d]*

[a] Institute for Ecopreneurship, School of Life Sciences, University of Applied Sciences and Arts North-western Switzerland, Hofackerstrasse 30, 4132 Muttenz, Switzerland

[b] EPFL, Institute of Materials Science and Engineering, Ecole Polytechnique Fédérale de Lausanne, Station 12, Lausanne, 1015, Switzerland

[c] Empa, Swiss Federal Laboratories for Materials Science and Technology, Laboratory for Functional Polymers, Dübendorf, 8600, Switzerland

[d] Department of Environmental Technology, Wageningen University, 6708 PB, Wageningen, the Netherlands

[e] Department of Physics, University of Oxford, Clarendon Laboratory, Parks Road, Oxford OX1 3PU, UK

*Email: markus.lenz@fhnw.ch

Contents

- **Table S1**: Composition of a PSC with ITO/sPACz/$FAPbI_3$/C_{60}-BCP/Ag device configuration.
- **Figure S1:** Acidic extraction of a synthetic PSC mixture
- **Section S1:** Acidic extraction of glass-based PSC
- **Figure S2:** Acid extraction of a glass-based PSC
- **Table S2:** Yields and Global Warming Potentials (GWP) per m^2 PSC for In and Ag extraction
- **Table S3:** Global Warming Potential (GWP100) per m^2 PSC of In (Yield 87.3%) and Ag (Yield 87.06%) leaching in 5% HNO_3 at various acid reuses
- **Section S2:** Life cycle assessment
- **Table S4:** The Life cycle inventory of acid extraction of In and Ag of recycled PSC at different experimental parameters, amounts for $1m^2$ PSC
- **Figure S3:** In and Ag speciation in 5% HNO_3
- **Figure S4:** Scheme of the LbL filtration setup
- **Section S3:** Calculation of the pressure-dependent energy consumption of a membrane filtration pump
- **Section S4:** Precipitation of In and Ag after separation by NF

39

Table S1: Composition of a PSC with ITO/sPACz/$FAPbI_3$/C_{60}-BCP/Ag device configuration. Components written in bold were considered for synthetic extraction. Reactors were scaled down depending on the experimental setup; typically, 20 mL was used as the total volume.

**PSC component**	**Layer functionality**	**Thickness (nm)**	**Density (g/cm^3)**	**Molar mass (g/mol)**	**Component concentration (mg/m^2 = mg/L**
**In_2O_3**	**TCO component**	**120 – 160**	**7.14**	**277.63**	**771.12 – 1028.16**
**SnO_2**				**150.71**	**85.68 – 114.24**
PEDOT:PSS	hole transport				
$FACsPbSnI_3$	perovskite absorber precursor	500 – 600	4.25	884.58	removed during hot water extraction[1]
C_{60}-BCP	electron transport				
**Ag**	**bottom electrode**	**100**	**10.49**	**107.87**	**268.5**

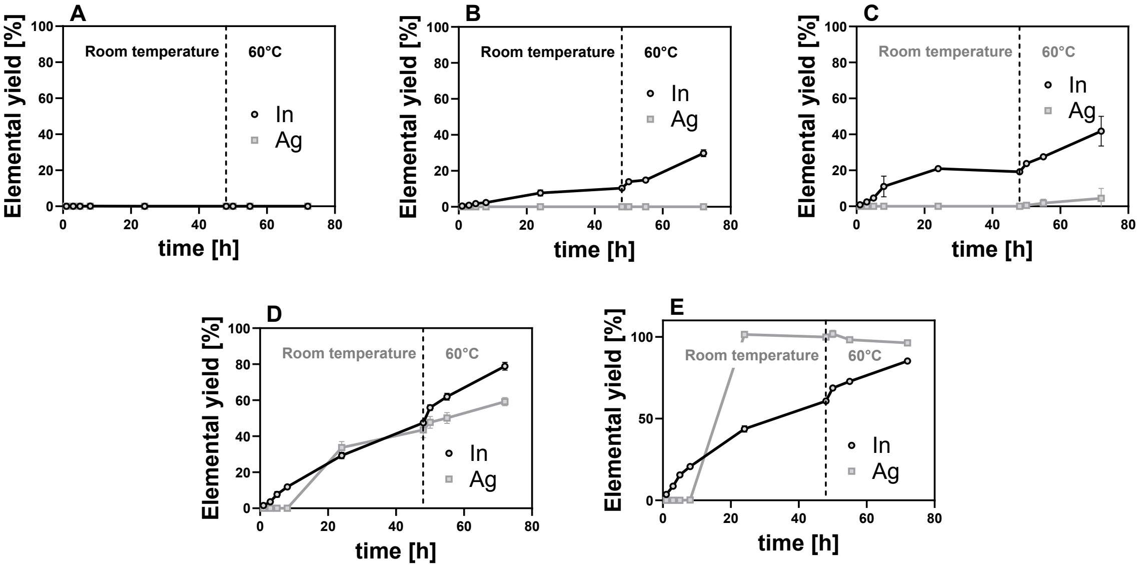

Figure S1: Acidic extraction of a synthetic PSC mixture using an area equivalent of $1L/m^2$ and deionized H_2O **(A)**, 1% HNO_3 **(B)**, 3% HNO_3 **(C)**, 5% HNO_3 **(D)** and 10% HNO_3 **(E)**.

Section S1: Acidic extraction of glass-based PSC

Acidic extraction of glass perovskite stacks (9 cm^2) using 5% HNO_3 and an area equivalent of 1 L/m^2 resulted in the extraction of 0.2 mg In and Ag, respectively, after 24h of extraction (Figure SI 2). After only 3 hours of extraction, 0.2 mg of the metal was extracted, and the metal concentration in the solution only marginally increased over the course of the extraction. When the acid volume was increased to 2 L/m^2, In and Ag extraction was considerably higher, resulting in 0.87 ± 1.4 mg In and 0.65 ± 0.16 mg Ag extracted after 24h.

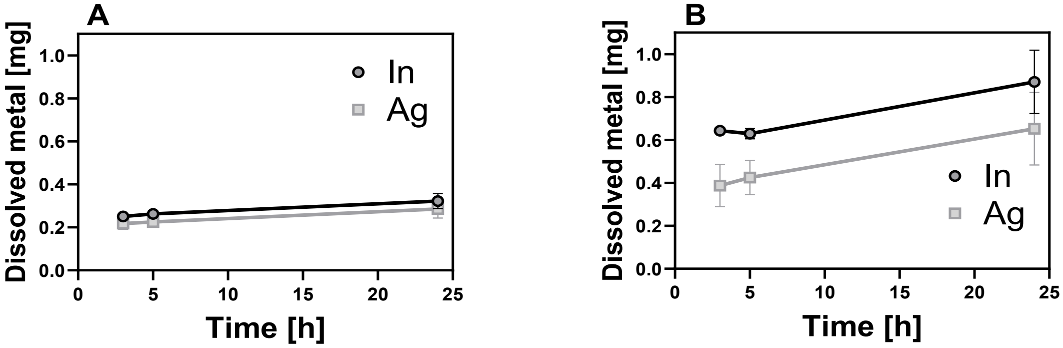

Figure S2: Acid extraction of a glass-based PSC using 5% HNO_3 with a volume to area equivalent of 1 L/m^2 **(A)** and 2 L/m^2 **(B).** Experiments were conducted in triplicates at 80˚C.

Table S2: Yields and Global Warming Potentials (GWP) per gram In and Ag extracted from PSC in 10% and 5% HNO_3 over time

	10% HNO_3				5% HNO_3			
**Time [h]**	**% In Dissolved**	**GWP (kg CO_2/g In)**	**% Ag Dissolved**	**GWP (kg CO_2/g Ag)**	**% In Dissolved**	**GWP (kg CO_2/g In)**	**% Ag Dissolved**	**GWP (kg CO_2/g Ag)**
1	21.31 ± 4.80	2.84 ± 0.48	86.50 ± 1.19	0.98 ± 0.13	8.15 ± 1.56	4.62 ± 0.05	81.60 ± 2.26	0.67 ± 0.10
2	39.01 ± 4.84	1.55 ± 0.22	85.49 ± 6.47	1.03 ± 0.13	18.65 ± 3.49	2.03 ± 0.02	83.51 ± 7.65	0.60 ± 0.09
3	53.11 ± 6.04	1.16 ± 0.16	89.03 ± 5.35	0.98 ± 0.13	29.01 ± 6.44	1.32 ± 0.02	83.76 ± 7.22	0.60 ± 0.09
5	71.38 ± 4.46	0.14 ± 0.06	92.98 ± 3.05	0.99 ± 0.11	42.91 ± 8.45	0.90 ± 0.01	84.97 ± 7.71	0.60 ± 0.09
7	82.61 ± 4.01	0.76 ± 0.10	89.43 ± 5.85	1.00 ± 0.13	57.26 ± 10.42	0.69 ± 0.01	84.75 ± 7.13	0.61 ± 0.10
24	91.94 ± 1.66	0.73 ± 0.10	89.82 ± 4.42	1.06 ± 0.15	87.30 ± 7.86	0.51 ± 0.01	87.06 ± 7.48	0.67 ± 0.12

Table S3: Global Warming Potential (GWP100) per gram In (Yield 87.30%) and Ag (Yield 87.06%) leaching in 5% HNO_3 at various acid reuses

**Number of acid volume uses**	**HNO_3 5% Volume (L)**	**GWP (kg CO_2/g In)**	**GWP (kg CO_2/g Ag)**
1	1	0.51	0.67
2	0.5	0.244	0.32

5	0.2	0.146	0.191
10	0.1	0.113	0.148

Section S2: Life cycle assessment

The goal of the LCA is to study the influence of experimental parameters (acid concentration, reuse cycles of the acid and process time) on the global warming potential (GWP 100)[2] in kg CO_2 equivalents per gram In and per gram Ag obtained by acid extraction from shredded PSC. The LCA was performed using the SimaPro software and the Ecoinvent v3 database. The system boundary focused on the acid and power consumption for the extraction of In and Ag at the conditions of the experiments. Previous process steps such as PSC shredding, and subsequent process steps such as filtration and waste disposal were excluded in this study. The Life cycle inventory (LCI) is listed in Table S4.

Table S4: The Life cycle inventory of acid extraction of In and Ag of recycled PSC at different experimental parameters, amounts for $1m^2$ PSC

**Flow**						
Process Time (h)	1	2	3	5	7	24
Electricity, medium voltage, region Europe (Wh)	14.40	14.85	15.30	16.21	17.11	24.80
Nitric acid 5% (L), without reuse	1	1	1	1	1	1

Nitric acid without water (g)	75	75	75	75	75	75
Softened water (g)	950	950	950	950	950	950
Nitric acid 10% (L), without reuse	1	1	1	1	1	1
Nitric acid without water (g)	150	150	150	150	150	150
Softened water (g)	900	900	900	900	900	900

The amount of acid refers to one experiment. The acid can be used in up to ten cycles (see also section 4.1), consequently the amount of acid in the LCI was divided by the number reuse cycle for the calculation of The GWP Electricity is used for agitation of the shredded PSC flakes with the acid and for heating the mixture. Towler and Sinnott[3] report electrical power of 1.0-1.5 kW/m^3 for medium-strength stirring of liquids. For the calculations we have considered the higher value. The heating energy is approximated by the energy required to heat water from 20˚C to 80˚C and to compensate a heat loss of 80 W/m^2 at the surface of an open process vessel with an area to volume ratio of 19.0 m^2/m^3 [3].

Since the output of the process are the two valuable metals which are in the same amounts in the PSC, the GWP of the process is allocated 50% to Ag and 50% to In.

The sensitivity of the GWPs obtained in the Life cycle Impact Assessment of experimental setups was analysed in a Monte Carlo Analysis considering a confidential interval of 68%.

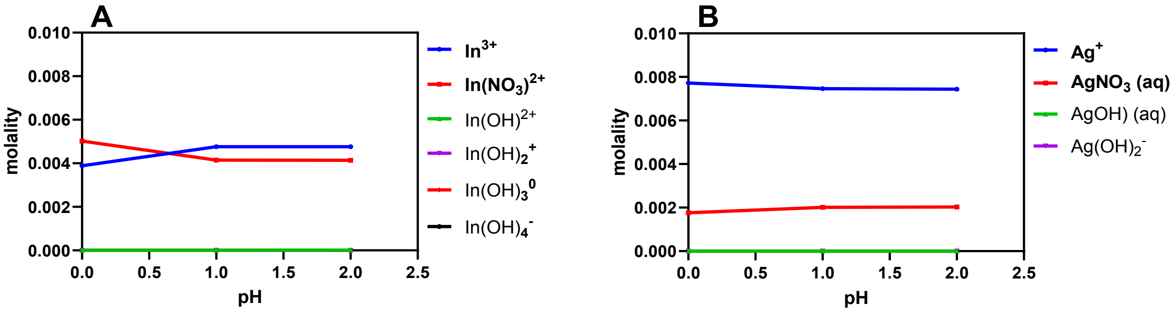

Figure S3: In **(A)** and Ag **(B)** speciation in 5% HNO_3 (data created with Geochemist Workbench®)

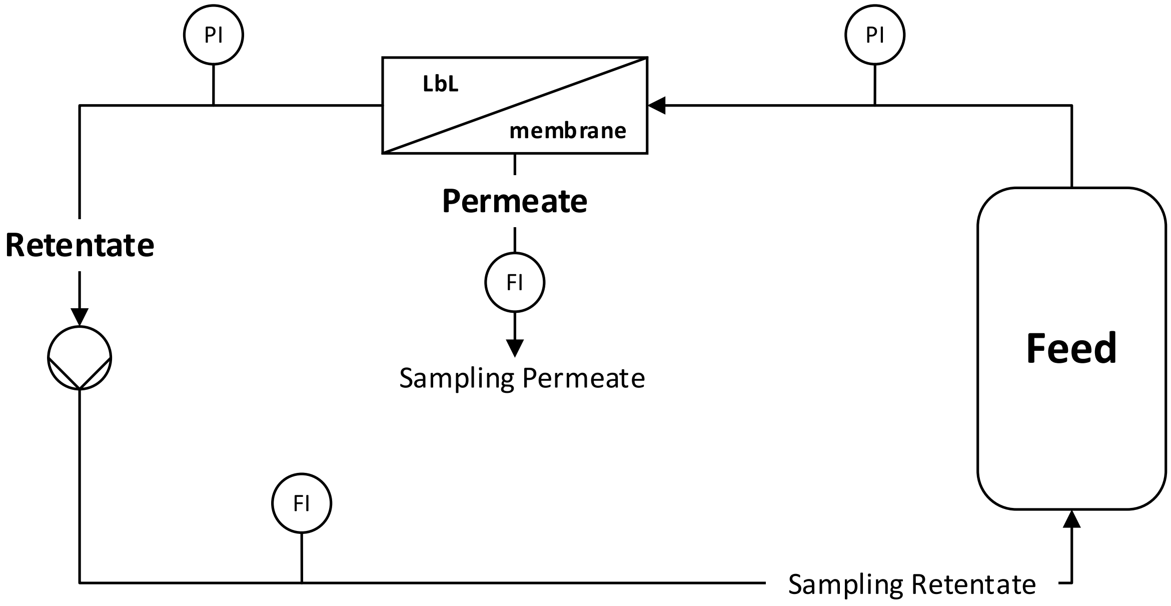

Figure S4: Scheme of the LbL filtration setup (PI: pressure sensor, FI: crossflow measurement)

Section S3: Calculation of the pressure-dependent energy consumption of a membrane filtration pump

The pressure-dependent power consumption of a membrane filtration pump was calculated using Equation 1

$$P\ [kW] = Q\left[\frac{L}{min}\right] \times p\ [bar] \times \frac{1}{\eta} \qquad \text{Eq. 1}$$

where Q represents the crossflow of the feed, p is the transmembrane pressure (TMP) and η is the pump efficiency (for “ideal” operation $\eta = 1$).

The energy consumption for membrane filtration was calculated with Equation 2

$$Q\ [kWh] = P\ [kW] \times t\ [h] \qquad \text{Eq. 2}$$

To compare the energy consumption of the commercial flat sheet membrane (AMS 3012) with the LbL membrane [PES(PAH/PSS)$_4$], a higher surface area of the LbL membrane was assumed. An estimated LbL membrane area of 200 cm^2 resulted in a 27-hollow-fibre module with a flow rate of 1.4 L/min and a TMP of 5 bar. This corresponded to the area of the flat sheet AMS 3012 NF membrane (200 cm^2, 2.5 L/m, 25 bar). When compared to the experimentally assessed results of an AMS 3012 NF membrane (200 cm^2, 2.5 L/min, 25 bar), the LbL system resulted in a 10x lower energy consumption than the AMS 3012 system (Figure 5a).

The pressure drop over the membrane was calculated with Darcy’s law [4], resulting in a pressure drop of 0.021 bar for a 120 cm hollow fibre module. To ensure comparable filtration conditions, the filtration needed to be operated with increased pressure to compensate for the pressure drop.

In order to achieve an estimated upscaling to 1 m^2, the length of the hollow fibres was increased to 120 cm, resulting in a membrane module of 331 hollow fibres. The estimated upscaling of the AMS 3012 membrane was based on standard data provided by the supplier. Here, the LbL system resulted in an 8x lower energy consumption than the AMS 3012 system (Figure 5b).

Section S4: Precipitation of In after separation by NF

Chemical consumption for metal precipitation was determined based on calculations and literature values. Indium precipitation is most commonly achieved by pH increase using NH_4OH, for which an increase to pH 7 has been reported to result in efficient In precipitation [5]. The pH of the unchanged feed solution (5% HNO_3 based) was measured to be pH 0, corresponding to 1 mol/L of free H^+. Hence, for neutralisation to pH 7 (c (H^+) = 10^{-7}), roughly 1 mol/L (17 g/L) NH_4OH would be required.

As the volume fraction of the In-containing retentate was 10 – 20%, a considerable reduction of NH_4OH consumption was possible (17.0 g/L → 3.4 g/L).

(1) Schmidt, F.; Amrein, M.; Hedwig, S.; Kober-Czerny, M.; Paracchino, A.; Holappa, V.; Suhonen, R.; Schäffer, A.; Constable, E. C.; Snaith, H. J.; Lenz, M. Organic Solvent Free PbI2 Recycling from Perovskite Solar Cells Using Hot Water. *Journal of Hazardous Materials* **2023**, *447*, 130829. https://doi.org/10.1016/j.jhazmat.2023.130829.

(2) Intergovernmental Panel On Climate Change (Ipcc). *Climate Change 2021 – The Physical Science Basis: Working Group I Contribution to the Sixth Assessment Report of the Intergovernmental Panel on Climate Change*, 1st ed.; Cambridge University Press, 2023. https://doi.org/10.1017/9781009157896.

(3) *Heat Loss from Open Water Tanks*. https://www.engineeringtoolbox.com/heat-loss-open-water-tanks-d_286.html (accessed 2024-09-20).

(4) Pacella, H. E.; Eash, H. J.; Frankowski, B. J.; Federspiel, W. J. Darcy Permeability of Hollow Fiber Bundles Used in Blood Oxygenation Devices. *Journal of Membrane Science* **2011**, *382* (1), 238–242. https://doi.org/10.1016/j.memsci.2011.08.012.

(5) Zheng, K.; Benedetti, M. F.; van Hullebusch, E. D. Recovery Technologies for Indium, Gallium, and Germanium from End-of-Life Products (Electronic Waste) – A Review. *Journal of Environmental Management* **2023**, *347*, 119043. https://doi.org/10.1016/j.jenvman.2023.119043.
